# Coronavirus Disease 2019 (COVID-19) Pandemic and Economic Impact

**DOI:** 10.12669/pjms.36.COVID19-S4.2638

**Published:** 2020-05

**Authors:** Tauseef Ahmad, Haroon, Mukhtiar Baig, Jin Hui

**Affiliations:** 1 Tauseef Ahmad, M.Phil. Department of Epidemiology and Health Statistics, School of Public Health, Southeast University, Nanjing 210009, China, Key Laboratory of Environmental Medicine Engineering, Ministry of Education, School of Public Health, Southeast University, Nanjing 210009, China; 2 Haroon, M.Phil. College of Life Science, Northwest University, Xian, China; 3 Dr. Mukhtiar Baig, Ph.D. Clinical Biochemistry, King Abdulaziz University, Jeddah, Saudi Arabia; 4 Dr. Jin Hui, Ph.D. Department of Epidemiology and Health Statistics, School of Public Health, Southeast University, Nanjing 210009, China, Key Laboratory of Environmental Medicine Engineering, Ministry of Education, School of Public Health, Southeast University, Nanjing 210009, China

**Keywords:** Coronaviruses, COVID-19, China, Economic losses

## Abstract

In less than two decades, the world has experienced three outbreaks of deadly Coronaviruses, including the recent pandemic of Coronavirus Disease 2019 (COVID-19) in China. COVID-19 posed an emergency of international concerns, and cases have been reported in more than 200 countries/regions that resulted in health, lives, and economic losses. China’s economic growth is projected to fall to 5.6% this year, the International Monetary Fund (IMF) projected that policy investment and tax policies to implement $3.3 trillion and contributes further $4.5 trillion. IMF forecasts grow from 3.7% of global gross domestic product (GDP) in 2019 to 9.9% in 2020. GDP ratio projected from 3.0% in 2019 to grow 10.7% in 2020, the US ratio expected to increase from 5.8% to 15.7%. France, Germany, Italy, Japan, and the United Kingdom (UK) each reported public sector funding programs totalling > 10% of their yearly GDP. There is a dire need for regional and international co-operation to extend hands to prevent further spreading of COVID-19.

## BACKGROUND

Health disasters, both communicable and non-communicable diseases, not only have global health impacts but, at the same time causing wide-ranging socioeconomic disruptions and losses. To avoid the international spread of disease and socioeconomic losses, identification of the biological threats is of great concern. Therefore, there is a dire need to strengthen national public health systems to achieve the above-stated goals.^1^,^2^ Coronaviruses cause several diseases in animals and humans.^3^ In the last two decades, Coronaviruses have caused three major outbreaks, including the on-going pandemic of Severe acute respiratory syndrome coronavirus 2 (SARS-CoV-2). The SARS-CoV-2 caused the disease known as Coronavirus Disease 2019 (COVID-19) and the first time emerged in China as an important threat to public health due to its association with an increasing number of infected cases and fatality. The Director-General of the World Health Organization (WHO) announced the novel coronavirus” or 2019-nCoV on February 11. Later on, Coronaviridae Study Group (CSG) of the International Committee on Virus Taxonomy that is the authority for the categorization of viruses and taxon nomenclature of the Coronaviridae family considered the placement of human pathogens, provisionally designated 2019-nCoV, within Coronaviridae. Based on phylogeny, taxonomy and existing procedure, the CSG accepts this virus as a variant of human and bat acute respiratory coronavirus syndrome (SARS-CoVs) of the species Severs acute respiratory coronavirus-related syndrome and labels it as SARS-CoV-2.^4^ The identification of the SARS-CoV-2, genome characterization (29,891-base pair) that shared 79.6% sequence identity to SARS-CoV BJ01 (GenBank accession number AY278488.2).^5^ Further details assessment and investigation are much needed at the national and international level to control and curb the disease. The WHO has announced, “the COVID-19 epidemic a public health emergency of international concerns.”^6^ WHO is committed to continuing to implement a comprehensive risk communication strategy to control the spreading of COVID-19 and enhance the public health procedures for repression of the current outbreak, safeguard the resilience of the health system and defend the healthcare personnel, and augment the investigations of the cases across China.^7^ The WHO and China are partners in investigations process and better understand the epidemiology and the advancement of the epidemic and measures, and share related information on human cases, and continues to recognize the zoonotic source of the epidemic, screening at international airports and ports and carry out further evaluation and treatment.^6^

Active community health members are required in each district for control and measurement of the outbreak. Scientists and researchers around the world are racing to find out more about the SARS-CoV-2, including how it spreads and information about its genetic sequences. Evolving the community in an intervention such as the closing of the main entries is a positive outcome that will help for prevention and control. Therefore, it brings hope to this outbreak among the community through increased community awareness are needed to be addressed.

In response to the outbreak on January 24, 2020, the Wuhan government proposed to build the Huoshan hospital to battle again SARS-CoV-2 within the ten days in Wuhan with a covered area of 34,000 square meters and provides 1000 beds which is great offers by the government in response to the outbreak.^8^ The efforts to respond to the situation on January 26, 2020, many organizations and institutions started working in developing vaccines based on the published genome.^9^ Throughout China, the people were restricted to their homes. The hospitals, health disk centers, screening points, and treatment centers provide their services to the local population.^10^ Therefore, there is a dire and urgent need to increase awareness and safety measures to stop the spreading of COVID-19. So far, the Government of China took the initiative to suspend the public transportation, buses, subways, and other routes of the entrance to Wuhan city (which is considered hub zone for COVID-19) sealed the seafood markets and fully functioned the health facilities 24 hours.

**Fig.1:**
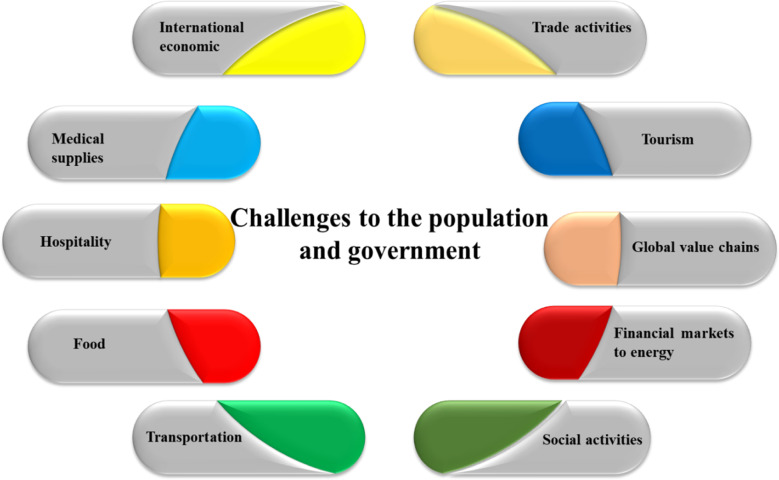
Challenges to the population and government.

Suspected, infected, and mortality ratio has crossed the previous outbreaks record by the viruses from the same family, such as SARS-CoV and MERS-CoV. It is worth mentioning that generally, it is believed that SARS and MERS were fatal, but the current figures have changed the scenario. As of 26 April 2020, 02:33 pm Nanjing Time, a total of 2,898,082 confirmed cases of COVID 19 has been reported globally, with 203,025 deaths.^11^ The COVID-19 infection spread so quickly that within the last two months and crossed the borders through the travellers and infected the people in more than 200 countries/regions across the globe. Despite lockdown and strict regulations regarding the COVID-19 outbreak, yet it has not been controlled, which shows that the transmission of this virus is much higher than other members of the same family. That’s why the number of deaths is much higher than previous outbreaks. Therefore, the local population and government were also facing some challenges to takeover on COVID-19 (Fig.1). Due to lockdown, isolation, and quarantine, people were facing problems regarding food, transportation, health, and social activities.

**Fig.2:**
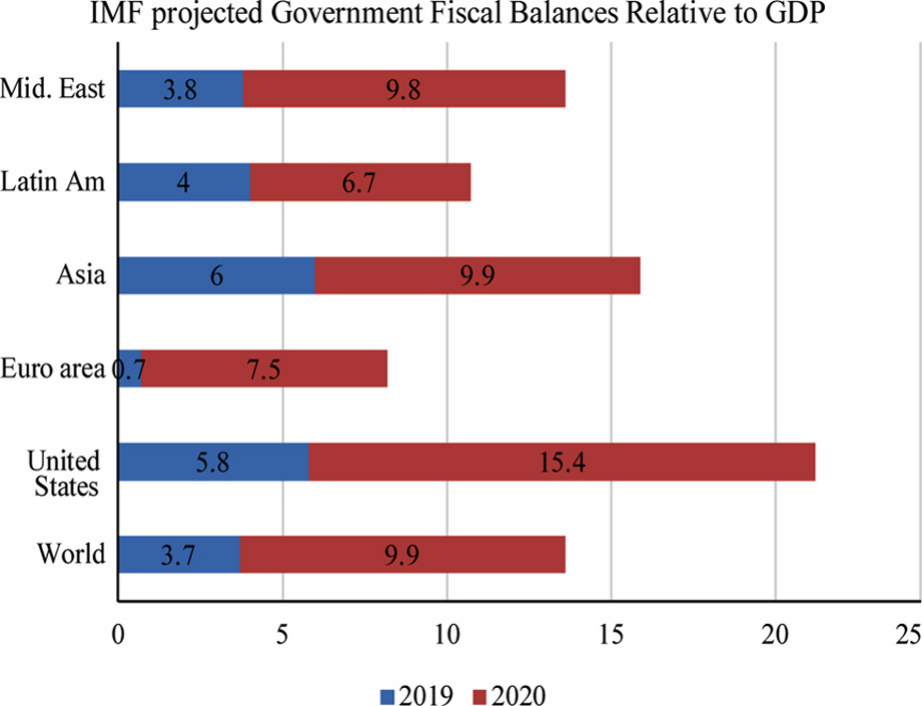
IMF projected Government Fiscal Balances Relative to GDP.^20^

**Fig.3:**
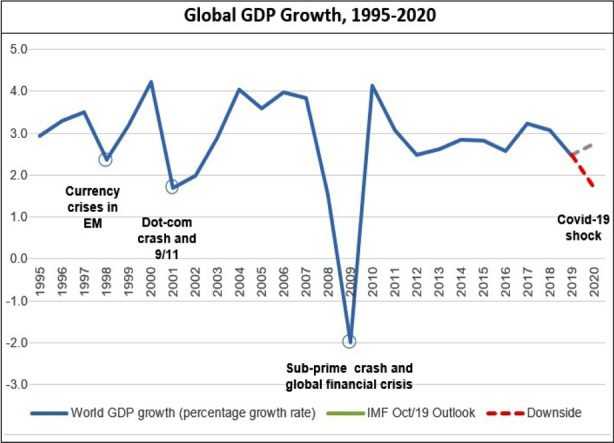
Global GDP growth, 1995-2020.^22^

The reported cases outside China have significantly associated with infected travellers to other countries. There is a dire need for international collaboration and regional co-operation to strengthen the public health system, and disease surveillance to control and stop further transmission of this deadly virus. The action was expected from WHO to implement rigorous roles and play an active role in making a special coordination cell that coordinates between all the countries where it has been detected. If strict actions have not been taken, then there is a chance of transmission of this coronavirus to other countries, and also the mortality rate will keep increasing.^12^ Some neighbouring countries of China with limited health facilities are at considerable risk of the future trajectory of COVID-19. Those countries are even unable to diagnose the virus rather than treating it at BSL-3 and BSL-4 as per protocol. This suggests that on every airport in China, travellers must be screened before they allow travelling nationally and internationally. The scientists and researchers are working hard to get a drug or vaccine against COVID-19.^13^

## ECONOMIC IMPACT

Strict restrictions on board on the movement of peoples from Wuhan city, Hubei province, and now expanded to other parts of China, prohibited business-related travelling as well as the movement of goods and workers.^14^ The human and economic cost of the coronavirus epidemic is escalating in China and beyond. Due to the SARS-CoV-2, government and private employees and customers stop entering their work, tourists and visitors complain that they may be exposed to the risk of infection. As a result, communities, mosques, churches, temples, restaurants, cinemas, transportations, hotels, sightseeing sites, big markets, and shops are all experiencing a rapid impact, which means that these sectors have been closed and particularly vulnerable to commercial losses. When a deadly, pneumonia-like virus known as Serve acute respiratory syndrome coronavirus in China in 2002, it affected not only the Chinese economy but also the economy of the other parts of the world.^15^ The extended closure of the Lunar New Year holidays has already caused havoc with internal travel, jobs, and even operations for many small businesses that can expect to feel the brunt of the pain. The confusion throughout the outbreak and the containment steps, in addition to the lack of enough workers, make billing and borrowing difficult for businesses operating off cash flow. The government has issued rules specifying that employees must be compensated on extended holidays or if they are unable to return to work due to quarantine restrictions.^16^ Several major industrial zones, including Shanghai, Suzhou, and Guangdong provinces, have prolonged their holidays for at least another week, stopping workers from returning. It is projected that China’s economic growth would drop to 5.6% this year from 6.1% last year, according to a conservative Oxford Economics forecast based on the virus impact so far. This would reduce global economic growth by 0.2% for the year to an annual rate of 2.3% the slowest since the global financial crisis a decade ago.^15^ Large numbers of airlines have cancelled flights to China, some of which are not expected to resume until the end of April. There are limited public health emergency restrictions in place and restricted flights to China; thus, the multinational companies’ activities in China are also expected to be confined. Asian tourism is also likely to be severely affected, particularly as neighbouring countries enforce strict travel bans on Chinese tourists, with losses estimated at $1.5 billion.^16^ The Chinese factories may affect, “drop orders for imported machinery, components and raw materials, computer chips from Taiwan and South Korea, copper from Chile and Canada, and factory equipment from Germany and Italy, auto factories in the American Midwest and Mexico to clothing plants in Bangladesh and Turkey.”^15^

The current outbreak affected Chinese tourism and beyond; similarly, five million Chinese tourists travelled to Vietnam in 2018, while Singapore hosted 3.4 million tourists from mainland during 2018, and the bushfire catastrophe has already stumbled the Australian economy. In the financial years 2018-19, 1.4 million Chinese tourists visited Australia. Chinese tourists’ visits to other countries have risen from 20 million in 2003 to 150 million in 2018. As a result, the susceptibility of several Asian-Pacific economies to a slowdown in Chinese tourist visits has increased significantly over the last two decades. “Thailand, Singapore, Malaysia, Vietnam, Hong Kong, Japan, South Korea, and Cambodia are among the most vulnerable Asian economies to the negative economic impact of Chinese tourism collapse”.^17^

When the WHO declared the pandemic to a high global health emergency, which causes a substantial negative effect on the growth of the global economy.^18^ International organizations have taken steps to offer loans and other financial aid to countries in need. The IMF projected that policy investment and tax policies to maintain economic growth implemented through mid-April 2020 amounted to $3.3 trillion and that grants, equity contributions and subsidies amounted to a further $4.5 trillion. Therefore, the IMF forecasts that the growth in borrowing by governments worldwide will grow from 3.7% of GDP in 2019 to 9.9% in 2020. For developing economies, the monetary balance-to-GDP ratio projected to grow from 3.0% in 2019 to 10.7% in 2020; the US ratio expected to increase from 5.8% to 15.7%.^19^ For developed economies, the fiscal balance-to-GDP ratio expected to increase from 4.8% to 9.1%. According to the IMF, France, Germany, Italy, Japan, and the UK, each reported public sector support programs totalling > 10% of their yearly GDP.^20^ For the global crisis response, the IMF initially announced $50 billion for low-income IMF countries, which received up to $10 billion rapid disbursing of emergency financing. Rapid Financing Facility (RFI) offers approximately $40 billion in financial strains in emerging markets for COVID-19.^21^

Policymakers have adopted unique strategies to tackle temporary crises without disruption in markets that can mitigate the effect of the virus itself. The international health crisis, which seems to have become a global trade and economic crisis, with a rapidly increasing effect on the world economy.^23^ World leaders postponed international gatherings, some nations have allegedly promoted conspiracy theories that are blameful in other countries, the pandemic affects global affairs.^24^

## TREATMENT

World-wide scientists focus on new COVID-19 drugs and vaccinations, and numerous companies have produced anti-viral medicines possibly use for COVID-19. With COVID-19 reported cases with more than 2.7 million around the world and continuing to rise, the scientists moving forward with vaccinations and therapies to slow down the pandemic and mitigate the harm. In persons with an outbreak, antivirals drugs targeted the virus, and they keep the virus from replicating, and on certain occasions, they inhibit invasion of cells. Dr. Robert Amler claims antivirals are useful drug resources for fighting COVID-19, as well as vaccinations. Remdesivir medication stopped the propagation of the virus, Kaletra drugs are anti-HIV and are scheduled for SARS-CoV-2 clinical trailing; Favipiravir used for influenza and also used on a trial basis in China against COVID 19, and Arbidol is antiviral drugs checked for COVID-19. Other drugs including monoclonal antibodies (trigger the immune system), blood plasma transfers (plasma contains antibodies), stem cells and immune suppressants (immune system release proteins called cytokines).^25^

## FUTURE CONSIDERATION

Over time death toll due to the COVID-19 keeps ascending. It has crossed all the boundaries and borders despite strict safety measures like lockdown of most affected cities, restriction in movements, use of proper masks, and so on. Yet, dozens of new cases are being reported daily. This situation has put the policymakers and health personal in trouble to control the fatal COVID-19 pandemic.

A study conducted by Bogoch et al. analyzed 20 different destinations to which people travel from or to Wuhan, the most affected city. Almost all those destinations/countries got cases of COVID-19.^26^ Most of the cases reported earlier in China had history from Wuhan. Therefore, it is recommended to identify the individuals who have the travel history of Wuhan during December 2019, and onward should be thoroughly screened before they get connected with other individuals. The Covid-19 pandemic will permanently alter our environment before it is regulated. We ought to adjust how we wash our faces, cover our coughs, greet everyone, and how near we are. We should reconsider the need for gatherings and conventions and allow connectivity as a public service such as mail. Our approach to reducing the effects of Covid-19, would, therefore, inevitably improve as we know more about the virus and the efficacy of specific treatments, but only because their situation will jeopardize our safety. Moreover, additional precautionary measures like least contact with the people and with the pets should be maintained. The animals or food which thought to be the source of this virus should be banned in the markets, and personal hygiene should be maintained in order to avoid deadly COVID-19 infection.

COVID-19 cannot travel on radio waves/mobile networks especially 5G mobile networks. Sunny or hot and cold weather, drinking alcohol, hot bath, hand dryers and UV lamps will not protect us from COVID-19.^27^ Human body temperature remains around 36.5°C to 37°C, while hot water and UV radiation can cause skin irritation. However, the excellent means of protection from COVID-19 is frequent handwashing, and avoid to touch mouth, nose, and eyes.

### Authors’ Contribution

**TA** designed and wrote the original draft of manuscript conceived.

**TA, H, MB, & JH** did review, editing and final approval of manuscript.
